# A core outcome set for clinical studies of adhesive small bowel obstruction

**DOI:** 10.1111/codi.16158

**Published:** 2022-06-03

**Authors:** Tripartite Gastrointestinal Recovery SBO Group, Matthew J. Lee, Stephen J. Chapman, Sue Blackwell, Robert Arnott, Richard P. G. ten Broek, Conor P. Delaney, Nagendra N. Dudi‐Venkata, Rebecca Fish, Daniel Hind, David G. Jayne, Katie Mellor, Anurag Mishra, Greg O’Grady, Tarik Sammour, Gabrielle Thorpe, Cameron I. Wells, Albert M. Wolthuis, Nicola S. Fearnhead, Samuel Adegbola, Sachin Ananth, Giulia Bagaglini, Andrew Beamish, Neil Bibby, Natalie S. Blencowe, Leo R. Brown, Joris P. Bulte, Julie Carver, Christopher P. Challand, Shirley Chan, Lindsey Chisholm, Daniel Clerc, Peter O. Coe, Daniel Cox, Alison Culkin, Sarah Daniels, Aleksander Dawidziuk, Amanda Dawson, Thomas M. Drake, Daniel J. Drayton, Sarah Duff, Eloy Espin‐Basany, Martin D. Evans, Mohammed Fakhrul‐Aldeen, Nigel Fisher, Sahara Fleetwood‐Beresford, Suzannah Forshaw, Jon Gani, Sandra Haddon, Jennie Han, Jack Helliwell, Philip Herrod, Marianne Hollyman, James Hopkins, Jovan Juloski, Celia Keane, Yick Ho Lam, Lisa Love, Aoife Lynch, Giles Major, Andrew Maw, Frank McDermott, Jamie McVeigh, Asif Mehraj, Monica Millan, Helen Mohan, Susan Moug, Maureen Naylor, Richard Parnell, Francesco Pata, Adam Peckham‐Cooper, Gianluca Pellino, Peter Pockney, Victoria K. Proctor, Arjun Rajagopalan, Jonathan Robinson, Martin Rutegård, Arin Saha, Kapil Sahnan, Adele E. Sayers, Leandro Siragusa, Neil J. Smart, David Swain, Julie Thompson, Linda Tutty, Peter G. Vaughan‐Shaw, Danilo Vinci, Ravi Vissapragada, Katharine R. Wheelband, Annabelle Williams, Mohammed U. Younis

**Affiliations:** ^1^ Department of Oncology and Metabolism, The Medical School University of Sheffield Sheffield UK

**Keywords:** adhesions, core outcome set, gastrointestinal recovery, perioperative care, small bowel obstruction

## Abstract

**Aim:**

Adhesive small bowel obstruction (ASBO) is a common surgical emergency condition. Research in the field is plentiful; however, inconsistency in outcome reporting makes comparisons challenging. The aim of this study was to define a core outcome set (COS) for studies of ASBO.

**Methods:**

The long list of outcomes was identified through systematic review, and focus groups across different geographical regions. A modified Delphi consensus exercise of three rounds was undertaken with stakeholder groups (patients and clinicians). Items were rated on a 9‐point Likert scale. Items exceeding 70% rating at 7–9 were passed to the consensus meeting. New item proposals were invited in round 1. Individualised feedback on prior voting compared to other participants was provided. An international consensus meeting was convened to ratify the final COS.

**Results:**

In round 1, 56 items were rated by 118 respondents. A total of 18 items reached consensus, and respondents proposed an additional 10 items. Round 2 was completed by 90 respondents, and nine items achieved consensus. In round 3, 80 surveys were completed; one item achieved consensus, and five borderline items were identified. The final COS included 26 outcomes, mapped to the following domains: Interventions, need for stoma, septic complications, return of gut function, patient reported outcomes, and recurrence of obstruction, as well as mortality, failure to rescue, and time to resolution.

**Conclusion:**

This COS should be used in future studies in the treatment of adhesive SBO. Further studies to define a core measurement set are needed to identify the optimum tools to measure each outcome.

**What does this paper add to the literature?**

There is heterogeneity in outcome reporting in studies of small bowel obstruction. This core outcome set proposes a list of key items to be reported in future studies.

## INTRODUCTION

Small bowel obstruction (SBO) is a very common emergency surgical condition; obstruction is the indication for surgery in just under half of the 25 000 patients undergoing emergency laparotomy in the UK [1]. SBO is the cause of death in 3.5 people per 100 000 population each year in high income countries and 1.8 deaths per 100 000 population in low and middle income countries [2]. The commonest cause of mechanical SBO is adhesions, usually from previous abdominal surgery, accounting for around 50% of all cases. Postoperative adhesions may affect up to 3.5% of patients by 20 years [3]. Adhesive SBO (ASBO) typically manifests with cramping abdominal pain, abdominal distension, intolerance to enteral intake and bilious vomiting. Patients are at risk of dehydration, renal failure, infection including aspiration pneumonia, malnutrition and poor wound healing [4]. One third of cases of ASBO require treatment with surgical intervention; the remaining cases settle with supportive management only [5]. Morbidity and mortality following treatment remains significant [5] with reported mortality of 7.2% at 90 days post surgery [6, 7].

The quality of randomised data and outcomes reporting in ASBO is limited [7, 8, 9]. The most recent trials in ASBO focus on the use of water soluble contrast agents [10], or laparoscopic surgery [11]. Given the challenges of delivering research in this area, it is important to maximise the utility of studies to support the literature. As studies focus on interventions in different parts of the patient pathway, the outcomes selected for reporting may differ, meaning that some key outcome data is not addressed. A systematic review of outcomes reported in trials and observational studies of ASBO identified 45 different outcome measures used across 51 studies [12]. Such variation in reporting of outcomes results in difficulties comparing results between studies and in implementation of research findings into clinical practice.

One approach to reducing variation in practice is to develop a “Core Outcome Set” (COS). A core outcome set is an agreed, standardised set of outcomes that should be measured, as a minimum, in all studies in a particular health area [13]. A COS is not intended to be restrictive as additional outcomes may be reported. Ensuring consistency in reported outcomes is important as thorough outcome reporting might avoid issues such as reporting bias [14], maximise information from each study, and reduce heterogeneity in subsequent meta‐analysis [15].

The aim of this study was to develop a COS for use in studies reporting outcomes of adult patients undergoing treatment for adhesive small bowel obstruction (ASBO).

## METHODS

The core outcome set was developed with reference to the COS‐STAD guidelines [16] and reported in line with the COS‐STAR guidelines [17]. Ethical approval for the study was secured from the University of Sheffield Research Ethics Committee (REF: 034049). A protocol for the study has previously been published [18]. Necessary variations to the protocol are presented in supplement 2 in Appendix S1

### Scope

The intended scope and relevance of the COS was defined as being for clinical studies (observational and interventional studies), of all interventions in the management of adult patients with ASBO.

### Steering group

An international steering group was convened to oversee the development of the COS. This included key stakeholders, specifically medical professionals, specialist nurses, methodologists, and patient representatives. The steering group included representation from the UK, Belgium, Netherlands, India, Australia, New Zealand, and the USA. These team members were identified through recent publications in the field [18]. The steering group members are listed in supplement 1 in Appendix S1.

### Patient and public involvement

Two patient representatives with relevant experience were recruited to the steering committee following study conception, and contributed to design, delivery and analysis. They recommended a protocol change that brought borderline items to the final consensus meeting. They also helped maintain engagement from patient participants during the Delphi voting rounds and highlighted the importance of the patient voice at the consensus meeting. The patient representatives contributed to the final manuscript. Patient and public involvement (PPI) representatives also helped to prepare a plain english summary of items to be voted upon (supplement 3 in Appendix S1).

### Generation of long list

The initial long list was generated from studies included in a recent systematic review [12]. The long list was discussed at a series of focus groups which were conducted with groups of patients, clinicians, and allied health professionals from different regions. The focus group meetings used a nominal group [19] approach to allow participants to propose additional items for inclusion in the long list. Several groups were conducted to allow for different time zones. Each exercise included only one type of stakeholder group and participation of different stakeholder groups for example, medical, allied health professional, or patient.

Following the focus groups, the long list was reviewed by the steering group to ensure clarity of items, and with lay members of the group to ensure that lay accessible language was used in the surveys. Where composite items were identified (e.g., scores) they were split into constituent items.

### Delphi consensus

A three round modified Delphi consensus was conducted [20]. Participants were recruited through promotion on Twitter, and society and professional group email lists. Each round was open for 6 weeks, with a gap of 2–4 weeks between voting rounds.

All participants were asked to complete an electronic consent form at registration. This captured the name, email address, and stakeholder respondent group (patient, clinician (doctor), allied healthcare professional (e.g., nurse, dietitian)). When round 1 opened, the access link to the survey was mailed out to participants. The Delphi survey was delivered using Google Forms (Palo Alto, CA).

In round 1, longlisted candidate outcomes were presented in a random order to avoid biasing responses based on sequence of items. Respondents were asked to rate the importance of items on a nine‐point Likert scale, with 1 being not important, and 9 being very important. All items had to be rated to complete the survey. At the end of round 1, respondents were encouraged to propose any additional outcomes not yet represented in the survey. Additional items were reviewed by the steering group at the end of round 1. They were judged to be either already represented in the set, reflecting treatment rather than an outcome, or appropriate for inclusion as new items for voting in round 2.

Rounds 2 and 3 presented the remaining longlisted items in a random order for each participant, using the same ranking system as round 1. Ratings of items were reviewed after the close of each round. Respondents received a copy of voting results, which included their vote, and how that compared to each panel of voters.

The inclusion of items to be discussed at the final consensus meeting was determined by a priori criteria. Items where 70% of ratings were 7–9 across the patient, clinician, and allied health professional groups were passed to the consensus meeting, as were items where 90% of any one group rated the item 7–9. Negative selected criteria were also employed where if any item was rated 1–3 by 70% of each panel or by 90% of a single panel, it was removed from the process. The same cutoffs were applied after rounds 2 and 3.

Only participants completing the prior round were eligible to participate in the subsequent round. At the end of round 3, participants were asked if they were interested in participating in the virtual consensus meeting.

### Sample size

There is no formal sample size for a Delphi exercise. A larger sample drawn from a range of geographical regions is desirable to ensure the most representative COS. A target of 100 respondents was set for the first round, with retention of >70% respondents from round to round.

### Amendment to planned protocol

Following round 3, items that had not met consensus were reviewed with patient representatives. The recommendation of the patient representatives was that items that had been rated 7–9 by >60% by each panel should be individually discussed at the consensus meeting and considered for inclusion on a yes/no basis. These outcomes were termed “borderline” items.

### Virtual consensus meeting

A virtual consensus meeting was held using the Blackboard Collaborate platform (Blackboard Inc). Purposive sampling of interested participants was performed to maximise geographical and stakeholder representation. Invitations were issued until 15 final delegates were confirmed. Electronic consent was taken prior to the meeting in the same way as for the Delphi process. The meeting was chaired by an independent chair with expertise in development of core outcome sets and COMET methodology.

The consensus participants reviewed “borderline” outcomes (>60% of voters rating of 7–9) and provided in/out votes on these. It then reviewed items that had achieved consensus for inclusion in the open voting rounds. This did not permit the removal of items but could clarify wording or presentation/grouping. The rest of the meeting focused on wording or grouping of items. The threshold for decisions to add borderline items or edit wording was set at 80%, to ensure that the expressed consensus from the prior voting rounds was not easily overruled.

### Additional analyses

The presence of sampling bias during the consensus meeting was explored by comparing Round 2 summary scores (medians) of participants who did and did not take part in the consensus meeting. Scores of ≤1 were considered acceptable.

## RESULTS

### Long listing of potential outcomes

**FIGURE 1:**
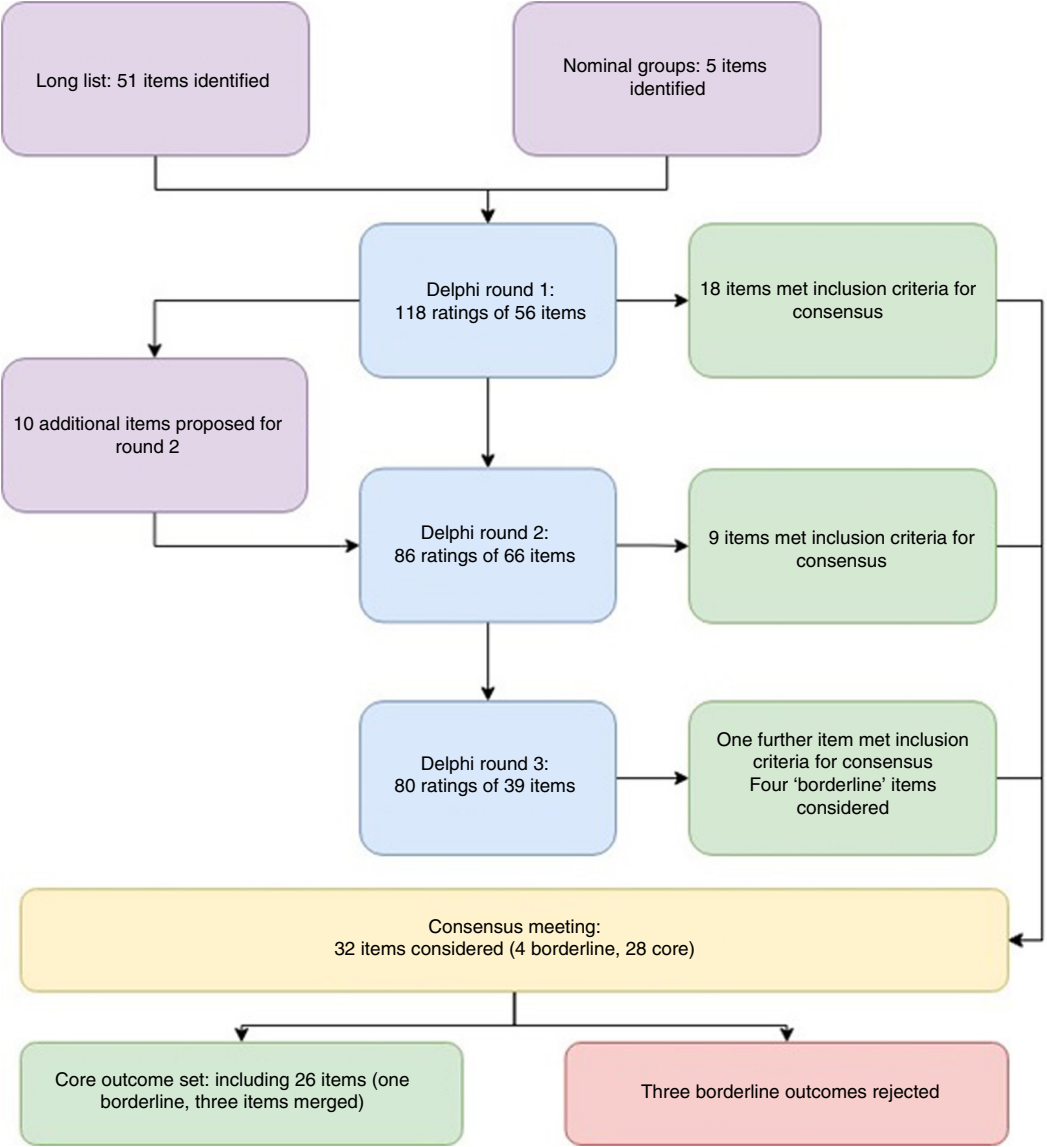
Flow of items through the study

Fifty‐one outcomes were extracted from the systematic review [12]. Five nominal group exercises were undertaken to explore additional potential outcomes to add to long‐listing of ASBO outcomes for consideration during Delphi consensus; three meetings were carried out in the UK separately with three patient participants, three clinician participants and two allied healthcare professionals. A further two nominal group discussions were undertaken with clinicians from Europe, each with three clinicians attending each session. This generated five additional items for the long list (2 from clinicians, 2 from patients, and 1 from allied healthcare professionals). The flow of items through the study is presented in Figure 1.

### Delphi round 1

Consent forms were completed by 201 potential candidates from all stakeholder groups, and 118 completed round 1 (58.7%). Characteristics of respondents and participation among stakeholder groups are presented in Table 1. This included 19 patients, 88 clinicians, and 11 allied health professionals. A total of 56 outcomes were considered for level of importance and 18 items reached a priori consensus for inclusion in definitive COS. Respondents proposed a further 30 outcomes. These were reviewed by the steering group, and 10 of these were added to the second round. Outcomes voted on in each round, along with the proportion of each panel rating them 7–9 are presented in Table S1.

**TABLE 1:** Summary of Delphi consensus participant characteristics

		Round 1	Round 2	Round 3
Participant type	Patient	19	14	14
	Clinician	88	67	61
	AHP	11	5	5
Participant location	Africa	4	1	‐
	Asia	1	3	3
	Australasia	18	12	10
	Europe	94	70	67
	North America	1	‐	‐
Retention rate	‐	‐	72.8%	93.0%

Abbreviation: AHP, allied health professionals.

### Delphi round 2

In round 2, 86 participants completed the survey. This included 14 patients, 67 clinicians, and five allied health professionals, reflecting 72.8% of those who completed round one. Respondents were asked to provide ratings on 48 items, 38 outcomes for reconsideration with feedback on voting patterns from the first round, and the 10 new outcomes added after round one. Of these 48 outcomes, nine met the a priori criteria for inclusion, and were carried to the consensus meeting.

### Delphi round 3

In round 3, 80 participants completed the survey. This included 14 patients, 61 clinicians, and five allied health professionals. This reflected 93.0% of respondents from round 2 and 68.6% of those who completed round 1. One further item was carried to the consensus meeting, and four borderline outcomes were identified.

### Consensus meeting

The consensus meeting was attended by four patient representatives, nine clinicians, and one allied health professional. One additional allied health professional withdrew due to personal circumstances. Analyses to assess for selection bias showed a median variation in ratings of 0, with no items showing variation of >1 (Supplement 4 in Appendix S1).

Four borderline outcomes were presented to the final consensus meeting. Of these, four were rejected by the group (votes for inclusion/exclusion presented in brackets with inclusion rate set at 80% consensus): complications, organ failure or injury (53.8%), incidence of pain (38.4%), length of hospital stay (23.1%), and stoma formation rate (7.7%). A fifth item “need for small bowel resection including new stoma” was initially rejected, but immediately reworded by the consensus group following feedback from the participants and accepted as an outcome “Need for small bowel resection with or without stoma” (100%). Discussion resulted in consensus merging of two short‐listed outcomes: “nonoperative management success rate” and “incidence of surgery after failed nonoperative management,” which were felt to reflect two facets of the same clinical outcome. These were merged into the single outcome “need for surgery following initial nonoperative management.” Another two outcomes, “recurrence‐free survival” and “obstruction recurrence rate” were merged by consensus into the outcome “timing and occurrence of further obstruction.” The remainder of the consensus meeting covered the presentation and arrangement of the COS, and discussion on the grouping of outcomes into domains. The consensus process resulted in a COS that included 26 items. These are presented in Table 2.

**TABLE 2:** Final core outcome set for adult patients experiencing adhesive small bowel obstruction (ASBO)

Domain	Outcomes
Need for and timing of interventions	Need for surgery following initial nonoperative management Time from admission to intervention
Need for small bowel resection	Need for small bowel resection with or without stoma Incidence of bowel strangulation
Septic complications	Systemic inflammatory response Peritonitis Sepsis Abdominal infection
Vomiting	Vomiting
Fluid balance	Overall fluid balance
Return of gut function	Time until resumption of a solid diet Postoperative ileus Time until relief of abdominal swelling/distension Time without adequate nutritional intake Time until return of bowel function
Resolution of obstruction	Time to resolution of obstruction
Patient reported outcomes experience	Patient satisfaction Return to normal activities of daily living
Return to theatre	Need for return to theatre
Complications	Morbidity
Mortality	Mortality
Failure to rescue	Failure to rescue
Readmission	Readmission
Recurrence of obstruction	Time until recurrence Timing and occurrence of further obstruction Recurrences needing surgery

## DISCUSSION

This study reports the development of a COS for studies investigating the treatment of patients with ASBO. It has included international engagement at all phases, and ensured representation from key stakeholders, especially patients, in all aspects. The COS for adults with ASBO presented here should therefore be widely acceptable and relevant across a range of healthcare systems.

The COS for ASBO highlights some of the differences between what is typically reported, and what is considered of importance to patients and their clinicians. Outcomes related to rates of surgical intervention, small bowel strangulation, and small bowel resection are reported in approximately 35%, 14%, and 6% of studies respectively [12], but rated consistently as highly important here. These are important markers of quality of care; surgery when indicated carries the risk of additional morbidity, while ischaemia and small bowel resection, that may be more commonly associated with delays to timely surgical intervention [6], are also associated with significantly increased rates of mortality [21]. Such outcomes are therefore relevant to patients and clinicians alike.

Markers of gastrointestinal recovery are also important in the context of ASBO, not just in the context of postoperative recovery but also in conservative management of ASBO. Time to resolution of bowel function was reported in 18% of prior studies, resumption of solid diet in 6%, and resolution in 2% [12]. These are all considered patient important factors in recently published qualitative work addressing recovery after abdominal surgery [22]. Failure of gastrointestinal recovery in both conservative and postoperative settings may result in malnutrition, that is associated with poor outcomes in ASBO [4]. A recent randomised trial examining operative approach in ASBO assessed several gastrointestinal recovery items as secondary outcomes [11], perhaps reflecting an evolving clinical recognition of this important part of a patient's recovery. In addition, fluid balance was also considered an important outcome. This has not been identified from any previous studies, but its relationship to dehydration, kidney injury, and general nutrition, is clinically apparent.

The timing of resolution of ASBO was included in the COS, having been reported in just 24% of previous studies, although a consistent definition was lacking. Previous studies have variously defined resolution as occurring when the patient had been discharged, factors related to passage of flatus, tolerance of diet, and use of radiological findings [12]. Clearly, there is work to be done to standardise this definition in the future.

Stakeholder participants also recognised that longer‐term outcomes are also important, specifically recurrence of SBO. Time to recurrence of SBO is variably reported, but is addressed in around two thirds of the reported studies [12], depending on how it is defined (recurrence‐free survival, 6%; whether recurrence occurred, 41%; or time to recurrence, 6%). The consensus meeting noted that recurrences requiring surgery were also important, as this is thought to affect up to 20% of patients, with significant impact on quality of life [23].

This COS is limited by a potential recruitment bias that may have favoured research‐active clinicians more involved in their professional associations and social media, and likewise favoured patients with access to internet, social media, patient peer support groups and technology. This has potentially excluded clinicians in middle and lower income countries, and patients in lower socioeconomic groups or who were frail or elderly. There was also a modest drop off in response rates between rounds 1 and 2. In the final consensus meeting, there were proportionately fewer allied health professionals and patients. This means that there may be an effect on the inclusion of borderline items. The final consensus was largely used to refine the COS and not remove the dimensions covered by any items, but the constitution of panellists may have influenced the final product.

The study has several strengths. It draws from a geographically broad set of respondents across all phases and ensures the patient voice is heard and prioritised throughout [24]. Long list generation was robust, drawing from multiple sources including published literature, nominal groups, and first round Delphi suggestions. Best practice methodology was used throughout, including randomisation of question order [25], and showing participants feedback from other stakeholder groups between rounds [26]. The use of 9‐point Likert scale allowed respondents greater distinction between the ratings of importance of items [27].

Adoption of the COS for adults with ASBO in future research will build a body of research data with consistent outcome reporting, allowing researchers to robustly compare treatments narratively, and through more reliable meta‐analysis [28]. Likewise, clinicians will be better able to translate research findings into clinical practice, and importantly, report outcomes that are important for patients. The authors recognise that implementation of a definitive COS into research practice may be difficult, but this has been readily achieved in areas such as rheumatoid arthritis, with uptake of a COS in around 80% of trials [29]. The authors will work with research funders, specialist associations, audit and registry managers, and the research community to ensure visibility of this COS and support its implementation.

## CONCLUSION

This study has identified 26 core outcomes to be reported in future studies where adhesive small bowel obstruction in adults is treated with conservative or surgical interventions. Implementation of this COS will promote consistent reporting and thereby improve the quality and comparability of research, as well as prioritising measurement of those outcomes of greatest importance to patients.

## CONFLICT OF INTEREST

The authors have no conflicts of interest to declare.

## ETHICAL STATEMENT

Ethical approval for the study was secured from the University of Sheffield Research Ethics Committee (REF:034049).

## Supporting information


Appendix S1

## Data Availability

All relevant data are presented in the article. No further data are available.
