# Supplementary material for: A core outcome set for clinical studies of adhesive small bowel obstruction
Source: Colorectal Dis. 2022 Jun 3;24(10):1204–10. doi: 10.1111/codi.16158 (PMC9796004; doi:10.1111/codi.16158)
Supplement: Supplementary file 1 — Appendix S1 [file CODI-24-1204-s001.docx]

**Supplementary table 1**

|  | **Round 1** | | | **Round 2** | | | **Round 3** | | |
| --- | --- | --- | --- | --- | --- | --- | --- | --- | --- |
|  | **Patients** | **Clinicians** | **Allied Health Professionals** | **Patients** | **Clinicians** | **Allied Health Professionals** | **Patients** | **Clinicians** | **Allied Health Professionals** |
| Recurrences needing surgery | 84.2% | 90.9% | 90.9% |  |  |  |  | |  |
| Intra-abdominal pressure gradient before and after intervention | 52.6% | 15.9% | 54.5% | 50.0% | 17.9% | 20.0% | 35.7% | 13.1% | 20.0% |
| Incidence of bowel strangulation | 89.5% | 92.0% | 90.9% |  |  |  |  |  |  |
| Time until recurrence | 94.7% | 77.3% | 90.9% |  |  |  |  |  |  |
| Time to resolution of obstruction | 89.5% | 92.0% | 100.0% |  |  |  |  |  |  |
| Complications: Cardiac | 47.4% | 38.6% | 36.4% | 71.4% | 46.3% | 40.0% | 71.4% | 39.3% | 20.0% |
| Non-operative management success rate | 94.7% | 88.6% | 100.0% |  |  |  |  |  |  |
| Complications: Thrombosis or embolism | 57.9% | 43.2% | 27.3% | 64.3% | 59.7% | 40.0% | 64.3% | 55.7% | 40.0% |
| Small bowel transit time | 63.2% | 30.7% | 63.6% | 57.1% | 17.9% | 40.0% | 50.0% | 27.9% | 40.0% |
| Admission to the intensive care unit | 57.9% | 78.4% | 72.7% | 64.3% | 76.1% | 80.0% | 50.0% | 77.1% | 80.0% |
| Gastric upset | 57.9% | 22.7% | 36.4% | 35.7% | 16.4% | 0.0% | 35.7% | 16.4% | 0.0% |
| Overall fluid balance | 84.2% | 54.5% | 72.7% | 85.7% | 58.2% | 100.0% |  |  |  |
| Mental wellbeing | 78.9% | 47.7% | 45.5% | 78.6% | 49.3% | 40.0% | 78.6% | 41.0% | 20.0% |
| Patient distress | 89.5% | 58.0% | 72.7% | 85.7% | 59.7% | 60.0% | 71.4% | 60.7% | 40.0% |
| Hospital re-admission rate | 89.5% | 80.7% | 81.8% |  |  |  |  |  |  |
| Time from admission to intervention | 89.5% | 85.2% | 90.9% |  |  |  |  |  |  |
| Time for gas canalization | 52.6% | 25.0% | 54.5% | 50.0% | 16.4% | 20.0% | 35.7% | 18.0% | 20.0% |
| Detection of contrast in the colon | 47.4% | 60.2% | 81.8% | 35.7% | 62.7% | 40.0% | 35.7% | 62.3% | 40.0% |
| Forced expiratory volume (1 s/forced vital capacity) | 36.8% | 13.6% | 18.2% | 57.1% | 9.0% | 0.0% | 57.1% | 8.2% | 20.0% |
| Incidence of morbidity | 84.2% | 87.5% | 90.9% |  |  |  |  |  |  |
| Complications: Sepsis | 84.2% | 73.9% | 72.7% | 85.7% | 80.6% | 80.0% |  |  |  |
| Xray/radiation burden | 47.4% | 25.0% | 36.4% | 35.7% | 17.9% | 0.0% | 42.9% | 13.1% | 0.0% |
| Complications: Organ injury or failure | 78.9% | 79.5% | 63.6% | 85.7% | 76.1% | 60.0% | 85.7% | 77.1% | 60.0% |
| Mortality | 73.7% | 92.0% | 100.0% |  |  |  |  |  |  |
| Incidence of surgery after failed non-operative management | 89.5% | 90.9% | 81.8% |  |  |  |  |  |  |
| Return to normal activities of daily living | 89.5% | 70.5% | 72.7% |  |  |  |  |  |  |
| Complications: Peritonitis | 73.7% | 77.3% | 81.8% |  |  |  |  |  |  |
| Incidence of pain | 78.9% | 58.0% | 72.7% | 64.3% | 61.2% | 80.0% | 71.4% | 65.6% | 60.0% |
| Daily nasogastric tube output | 47.4% | 61.4% | 81.8% | 50.0% | 59.7% | 80.0% | 35.7% | 60.7% | 60.0% |
| Duration of intravenous therapy | 73.7% | 53.4% | 81.8% | 78.6% | 56.7% | 80.0% | 28.6% | 52.5% | 80.0% |
| Radiological intestinal dilatation | 63.2% | 40.9% | 72.7% | 71.4% | 26.9% | 60.0% | 35.7% | 23.0% | 60.0% |
| Complications: Respiratory | 47.4% | 47.7% | 36.4% | 64.3% | 56.7% | 40.0% | 57.1% | 63.9% | 20.0% |
| Weight loss | 63.2% | 43.2% | 81.8% | 50.0% | 44.8% | 60.0% | 42.9% | 39.3% | 60.0% |
| Time until resumption of a solid diet | 47.4% | 67.0% | 90.9% | 35.7% | 49.3% | 80.0% |  |  |  |
| Duration of radiation exposure | 31.6% | 18.2% | 18.2% | 28.6% | 11.9% | 0.0% | 42.9% | 11.5% | 0.0% |
| Complications: Vomiting | 84.2% | 61.4% | 90.9% |  |  |  |  |  |  |
| Complications: Urinary | 63.2% | 19.3% | 36.4% | 57.1% | 19.4% | 20.0% | 50.0% | 14.8% | 0.0% |
| Suitability and tolerability of contrast media | 52.6% | 42.0% | 27.3% | 42.9% | 31.3% | 60.0% | 28.6% | 16.4% | 20.0% |
| Complications: Renal | 57.9% | 55.7% | 54.5% | 78.6% | 58.2% | 60.0% | 71.4% | 59.0% | 40.0% |
| Intensive care unit length of stay | 47.4% | 61.4% | 72.7% | 71.4% | 58.2% | 60.0% | 64.3% | 70.5% | 40.0% |
| Nasogastric tube placement duration | 63.2% | 70.5% | 72.7% | 78.6% | 67.2% | 80.0% | 50.0% | 70.5% | 80.0% |
| Complications: Pneumonia | 47.4% | 58.0% | 45.5% | 78.6% | 62.7% | 60.0% | 71.4% | 62.3% | 20.0% |
| Patient satisfaction | 94.7% | 68.2% | 45.5% |  |  |  |  |  |  |
| Time until abdominal radiograph improvement | 47.4% | 19.3% | 45.5% | 50.0% | 16.4% | 20.0% | 28.6% | 8.2% | 20.0% |
| Duration of hospital stay | 52.6% | 71.6% | 81.8% | 78.6% | 65.7% | 60.0% | 64.3% | 67.2% | 60.0% |
| Recurrence-free survival | 100.0% | 81.8% | 90.9% |  |  |  |  |  |  |
| Time until resumption of a liquid diet | 52.6% | 63.6% | 81.8% | 64.3% | 56.7% | 60.0% | 50.0% | 67.2% | 60.0% |
| Complications: Abdominal infection | 78.9% | 63.6% | 63.6% | 78.6% | 67.2% | 80.0% | 78.6% | 77.1% | 80.0% |
| Obstruction recurrence rate | 94.7% | 84.1% | 81.8% |  |  |  |  |  |  |
| Systemic inflammatory response | 68.4% | 53.4% | 63.6% | 92.9% | 53.7% | 60.0% |  |  |  |
| Time until resolution of pain | 84.2% | 56.8% | 63.6% | 71.4% | 56.7% | 80.0% | 71.4% | 49.2% | 40.0% |
| Complications: ileus | 73.7% | 69.3% | 90.9% |  |  |  |  |  |  |
| Total number of treatments | 84.2% | 65.9% | 72.7% | 100.0% | 73.1% | 80.0% |  |  |  |
| Microbiological measurement of bacterial translocation | 73.7% | 38.6% | 63.6% | 64.3% | 26.9% | 40.0% | 64.3% | 24.6% | 20.0% |
| Time until return of bowel function | 84.2% | 78.4% | 90.9% |  |  |  |  |  |  |
| Time until relief of abdominal swelling/distension | 63.2% | 45.5% | 81.8% | 71.4% | 43.3% | 100.0% |  |  |  |
| Healthcare costs (to health system) |  |  |  | 42.9% | 52.2% | 60.0% | 28.6% | 55.7% | 60.0% |
| Duration of total parenteral nutrition |  |  |  | 57.1% | 67.2% | 80.0% | 57.1% | 73.8% | 80.0% |
| Need for small bowel resection |  |  |  | 92.9% | 88.1% | 100.0% |  |  |  |
| Complications: incisional hernia |  |  |  | 78.6% | 44.8% | 20.0% | 71.4% | 39.3% | 0.0% |
| Complications arising from parenteral or enteral feeding |  |  |  | 42.9% | 67.2% | 80.0% | 57.1% | 65.6% | 60.0% |
| Failure to rescue |  |  |  | 78.6% | 83.6% | 80.0% |  |  |  |
| Stoma formation rate |  |  |  | 78.6% | 79.1% | 60.0% | 64.3% | 80.3% | 60.0% |
| Time to return of appetite |  |  |  | 35.7% | 26.9% | 80.0% | 21.4% | 31.2% | 60.0% |
| Incidence of hypokalaemia |  |  |  | 57.1% | 38.8% | 20.0% | 50.0% | 39.3% | 0.0% |
| Time without adequate nutritional intake |  |  |  | 78.6% | 80.6% | 100.0% |  |  |  |

Proportion of panels rating each item 7-9 in each round. Items blacked out in round one were added following round one. Items blacked out in round three had achieved consensus in round two.

**Supplement 1: Authors and Collaborators**

MJ Lee*^1,2^ , SJ Chapman*^3^ , S Blackwell^4^ , R Arnott^5^ , RPG ten Broek^6^ , CP Delaney^7^ , NN Dudi-Venkata^8^ , R Fish^910^ , D Hind^11^ , DG Jayne^3^ , K Mellor^12^ , A Mishra^13^, G O’Grady^14^ , T Sammour^15^ , G Thorpe^16^ , CI Wells^14^ , AM Wolthuis ^17^ , NS Fearnhead^18^ on behalf of the Tripartite Gastrointestinal Recovery SBO Group

Affiliations:

1. Department of Oncology & Metabolism, Medical School, University of Sheffield, Sheffield, UK

2. Sheffield Teaching Hospitals NHS Foundation Trust, Sheffield, UK

3. Leeds Institute of Medical Research, University of Leeds, Leeds, UK

4. Patient Representative, Liverpool, UK

5. Patient Representative, Green Templeton College, Oxford, UK

6. Department of Surgery, Radboud University Medical Center, Nijmegen, NL

7. Department of Colorectal Surgery, Cleveland Clinic, Cleveland, USA

8. Faculty of Health &amp; Medical Science, School of Medicine, University of Adelaide, Adelaide, AU

9. Colorectal and Peritoneal Oncology Centre, The Christie NHS Foundation Trust, Manchester, UK

10. Division of Cancer Sciences, University of Manchester, UK

11. Clinical Trials Research Unit, University of Sheffield, Sheffield, UK

12. Nuffield Department of Orthopaedics, Rheumatology and Musculoskeletal Sciences, University of Oxford, Oxford, UK

13. Department of Surgery, Maulana Azad Medical College, New Delhi, India

14. Department of Surgery, Faculty of Medicine and Health Sciences, University of Auckland, Auckland, NZ

15. Colorectal Unit, Department of Surgery, Royal Adelaide Hospital, Adelaide, AU

16. Faculty of Medicine & Health Sciences, University of East Anglia Norwich, UK

17. Department of Abdominal Surgery, University Hospital Leuven, Leuven, BE

18. Cambridge University Hospitals NHS Foundation Trust, Cambridge, UK

* Notes that MJL and SJC are joint first authors

**Contributions:** SJC, MJL, and NSF conceptualised the study and all named authors contributed to the design of the study, data analysis, and preparation of the manuscript. SB and RA provided patient insights and led the public involvement strategy. RF chaired the consensus meeting. The GI Recovery SBO collaborators contributed to all Delphi rounds, providing context and input on the final COS. All have seen and approved the final manuscript. MJL is the study guarantor.

**The GI Recovery SBO collaborators**

Adegbola S, Ananth S, Bagaglini G, Beamish A, Bibby N, Blencowe NS, Brown LR, Bulte JP, Carver J, Challand CP, Chan S, Chapman SJ ,Chisholm L, Clerc D, Coe PO, Cox D, Culkin A, Daniels S, Dawidziuk A, Dawson A, Drake TM, Drayton DJ, Duff S, Espin-Basany E, Evans MD, Fakhrul-Aldeen M, Fisher N, Fleetwood-Beresford S, Forshaw S,Gani J, Haddon S, Han J, Helliwell J, Herrod P, Hollyman M, Hopkins J, Juloski J, Keane C, Lam YH, Love L, Lynch A, Major G, Maw A, McDermott F, McVeigh J, Mehraj A, Millan M, Mohan H, Moug S, Naylor M, Parnell R, Pata F ,Peckham-Cooper A, Pellino G, Pockney P ,Proctor VK, Rajagopalan A, Robinson J, Rutegård M, Saha A, Sahnan K, Sayers A, Siragusa L, Smart NJ, Swain D, Thompson J, Tutty L, Vaughan-Shaw PG, Vinci D, Vissapragada R, Wells C, Wheelband KR, Williams A, Younis MU.

**Author ORCiDs**SJ Chapman: 0000-0003-2413-5690

MJ Lee: 0000-0001-9971-1635

S Blackwell: 0000-0002-2819-3727

R Arnott: Not applicable

RPG ten Broek: 0000-0002-4012-7968

CP Delaney: 0000-0002-7042-8067

NN Dudi-Venkata: 0000-0002-9775-3599

R Fish: 0000-0002-4239-6993

D Hind: 0000-0002-6409-4793

DG Jayne: 0000-0002-8725-3283

K Mellor: 0000-0002-4054-5975

A Mishra: 0000-0002-2302-0632

G O’Grady: 0000-0002-5998-1080

T Sammour: 0000-0002-4918-8871

G Thorpe: 0000-0002-0639-4229

CI Wells: 0000-0001-9221-6674

AM Wolthuis: 0000-0002-1200-387X

N Fearnhead: 0000-0002-8651-5886

**Supplementary File 2: Supplementary Methods**

*Protocol Variations*

In 2020, coronavirus disease 2019 (COVID-19) caused by the severe acute respiratory syndrome coronavirus 2 was declared as a global pandemic by the World Health Organisation. Social distancing regulations enforced by national legislatures precluded the planned face-to-face consensus meeting due to take place in Auckland, New Zealand in November 2020. Following extensive consultation within the Steering Committee, a decision was made to convert the consensus meeting to a virtual event delivered via an online teleconference service. Care was taken to ensure inclusive and fair participation throughout the event by means of a dedicated event facilitator (MJL) and independent chairperson (RF). A substantial amendment to the approved protocol was confirmed on 11^th^ January 2021 by the University of Sheffield Research Ethics Committee prior to the online consensus event (6^th^ March 2021).

In contrast to the published protocol (first online 30^th^ November 2019), but prior to the start of data collection (17^th^ January 2020), the Delphi process criteria for consensus were amended. This was done following careful consideration between members of the Steering Committee and with close involvement of patient representatives. Previous criteria for consensus were defined as ≥70% of participants in each stakeholder group assigning a score of 7-9 or ≤15% of participants in each group indicating a score of 1-3. The latter component of these criteria was considered to be insufficiently discriminative and was removed. An additional criterion for consensus (≥90% in a single key stakeholder group) was set to recognise outcomes that were of strong importance to individual groups and in particular to patients.

**Supplement 3: Plain English**

| Outcome | Plain English Summary |
| --- | --- |
| Mortality | Death |
| Incidence of morbidity | If you become ill after your operation as a result of the obstruction |
| Hospital re-admission rate | Whether you have to go back to hospital for the same problem |
| Intra-abdominal pressure gradient before and after intervention | Pressure inside your belly before and after treatment |
| Obstruction recurrence rate | Whether you have another episode of small bowel obstruction |
| Incidence of surgery after failed non-operative management | If you need an operation after non-surgical treatment has failed |
| Recurrences needing surgery | If you have a recurring obstructions needing surgery |
| Recurrence-free survival | How long it is before you have another obstruction |
| Time until recurrence | How long between leaving hospital and the next time you have an obstruction |
| Time until return of bowel function | How long it takes until your bowel starts to work again |
| Time until resumption of a liquid diet | How long it takes before you can drink fluids |
| Time until resumption of a solid diet | How long it takes before you can eat solid food |
| Small bowel transit time | How quickly food/fluids move from the start of the gut to the end |
| Forced expiratory volume (1 s/forced vital capacity) | How well you can exhale/breathe out |
| Incidence of pain | If you are in pain |
| Time until resolution of pain | How long it takes for the pain to stop |
| Patient satisfaction | How happy you are with your treatment |
| Patient distress | How distressed you are |
| Gastric upset | If you feel like you have an upset stomach |
| Daily nasogastric tube output | How much fluid per day is coming out of a tube up your nose |
| Nasogastric tube placement duration | How long you have to have a tube up your nose |
| Detection of contrast in the colon | After drinking a specialised drink, is it visible in your bowel on an xray scan |
| Duration of hospital stay | How long you have to stay in hospital |
| Time to resolution of obstruction | How long it takes for the obstruction to unblock |
| Time until abdominal radiograph improvement | How long until your gut looks better on an xray scan |
| Microbiological measurement of bacterial translocation | If germs have spread outside of the bowel |
| Time for gas canalization | The time it takes for your bowel to open as seen on a scan |
| Systemic inflammatory response | How inflammed your bowel and other tissues are |
| Suitability and tolerability of contrast media | Whether you are able to drink an oral contrast drink |
| Time until relief of abdominal swelling/distension | How long it takes for swelling of the belly to get better |
| Time from admission to intervention | How long it is between admission to hospital and start of treatment |
| Non-operative management success rate | If treatment without an operation is successful |
| Incidence of bowel strangulation | If your bowel becomes twisted, blocking its blood supply |
| Duration of radiation exposure | How long you are exposed to radiation from scans |
| Total number of treatments | How many treatments you have for your obstruction |
| Admission to the intensive care unit | If you need to go to the intensive care unit |
| Intensive care unit length of stay | How long you stay in the intensive care unit |
| Duration of intravenous therapy | How long you need to have fluids or medication via a drip/feeding line |
| Complications: Urinary | Problems with the bladder |
| Complications: Respiratory | Problems with the lungs |
| Complications: Pneumonia | A chest infection |
| Complications: Organ injury or failure | Organ failure |
| Complications: Thrombosis or embolism | Blood clots in the legs or lungs |
| Complications: Renal | Problems with the kidneys |
| Complications: Sepsis | Severe infection (septicaemia) |
| Complications: Cardiac | Problems with the heart |
| Complications: Abdominal infection | Infection inside the belly |
| Complications: Peritonitis | Inflammation inside the belly |
| Complications: Vomiting | Vomiting |
| Complications: ileus | The bowel "going to sleep" |
| Return to normal activities of daily living | How long until you feel well enough to do your normal daily routine/activities |
| Weight loss | How much weight you have lost |
| Mental wellbeing | How well you feel in yourself/mental health |
| Radiological intestinal dilatation | How swollen your intestines look on a scan |
| Overall fluid balance | How hydrated (or dehydrated) you are |
| Xray/radiation burden | How much radiation you receive in total from xray scans |
| Healthcare costs (to health system) | How much treatment costs the healthcare system |
| Duration of total parenteral nutrition | How long you need to be given liquid food via a drip/feeding line |
| Need for small bowel resection | If you need an operation to remove the obstructed part of bowel |
| Complications: incisional hernia | Protrusion of fat/bowel through the wound after the operation |
| Complications arising from parenteral or enteral feeding | Complications occuring as a result of liquid food/feeding line |
| Failure to rescue | Death following a recognisable/treatable complication |
| Stoma formation rate | If you need a colostomy/ileostomy as a result of the operation |
| Time to return of Appetite | How long it takes after the operation to feel like you want to eat |
| Incidence of hypokalaemia | If you develop low potassium levels (a salt found in the blood) |
| Time without adequate nutritional intake | Amount of time without sufficient food/nutrition intake |

**Supplement 4: Attrition Bias**

| **Outcome** | **Median rating in round 2 by consensus participants (n=14)** | **Median rating in round 2 by consensus non-participants (n=74)** | **Difference** |
| --- | --- | --- | --- |
| Recurrences needing surgery | 9 | 8 | 1 |
| Intra-abdominal pressure gradient before and after intervention | 5 | 5 | 0 |
| Incidence of bowel strangulation | 9 | 8 | 1 |
| Time until recurrence | 7.5 | 7 | 0.5 |
| Time to resolution of obstruction | 7.5 | 8 | -0.5 |
| Complications: Cardiac | 7 | 6 | 1 |
| Non-operative management success rate | 8 | 8 | 0 |
| Complications: Thrombosis or embolism | 7 | 7 | 0 |
| Small bowel transit time | 5.5 | 5 | 0.5 |
| Admission to the intensive care unit | 8 | 8 | 0 |
| Gastric upset | 5 | 5 | 0 |
| Overall fluid balance | 7 | 7 | 0 |
| Mental wellbeing | 7 | 7 | 0 |
| Patient distress | 7 | 7 | 0 |
| Hospital re-admission rate | 8 | 8 | 0 |
| Time from admission to intervention | 8 | 8 | 0 |
| Time for gas canalization | 4.5 | 4 | 0.5 |
| Detection of contrast in the colon | 6.5 | 7 | -0.5 |
| Forced expiratory volume (1 s/forced vital capacity) | 5.5 | 5 | 0.5 |
| Incidence of morbidity | 8 | 8 | 0 |
| Complications: Sepsis | 8 | 8 | 0 |
| Xray/radiation burden | 6 | 5 | 1 |
| Complications: Organ injury or failure | 9 | 8 | 1 |
| Mortality | 9 | 9 | 0 |
| Incidence of surgery after failed non-operative management | 9 | 8 | 1 |
| Return to normal activities of daily living | 7 | 7 | 0 |
| Complications: Peritonitis | 8 | 8 | 0 |
| Incidence of pain | 7 | 7 | 0 |
| Daily nasogastric tube output | 7 | 7 | 0 |
| Duration of intravenous therapy | 7 | 7 | 0 |
| Radiological intestinal dilatation | 6 | 6 | 0 |
| Complications: Respiratory | 7 | 7 | 0 |
| Weight loss | 6 | 6.5 | -0.5 |
| Time until resumption of a solid diet | 6 | 7 | -1 |
| Duration of radiation exposure | 5 | 5 | 0 |
| Complications: Vomiting | 8 | 7 | 1 |
| Complications: Urinary | 6 | 5 | 1 |
| Suitability and tolerability of contrast media | 5.5 | 6 | -0.5 |
| Complications: Renal | 7 | 7 | 0 |
| Intensive care unit length of stay | 7 | 7 | 0 |
| Nasogastric tube placement duration | 7 | 7 | 0 |
| Complications: Pneumonia | 7 | 7 | 0 |
| Patient satisfaction | 7 | 7 | 0 |
| Time until abdominal radiograph improvement | 5 | 4.5 | 0.5 |
| Duration of hospital stay | 7 | 7 | 0 |
| Recurrence-free survival | 7.5 | 8 | -0.5 |
| Time until resumption of a liquid diet | 7 | 7 | 0 |
| Complications: Abdominal infection | 8 | 7 | 1 |
| Obstruction recurrence rate | 9 | 8 | 1 |
| Systemic inflammatory response | 7 | 7 | 0 |
| Time until resolution of pain | 7 | 7 | 0 |
| Complications: ileus | 8 | 7 | 1 |
| Total number of treatments | 7.5 | 7 | 0.5 |
| Microbiological measurement of bacterial translocation | 5 | 6 | -1 |
| Time until return of bowel function | 7 | 8 | -1 |
| Time until relief of abdominal swelling/distension | 7 | 6.5 | 0.5 |
| Healthcare costs (to health system) | 6 | 7 | -1 |
| Duration of total parenteral nutrition | 7 | 7 | 0 |
| Need for small bowel resection | 9 | 8 | 1 |
| Complications: incisional hernia | 6 | 6.5 | -0.5 |
| Complications arising from parenteral or enteral feeding | 6.5 | 7 | -0.5 |
| Failure to rescue | 9 | 9 | 0 |
| Stoma formation rate | 7.5 | 8 | -0.5 |
| Time to return of appetite | 6 | 6 | 0 |
| Incidence of hypokalaemia | 6 | 6 | 0 |
| Time without adequate nutritional intake | 7 | 8 | -1 |
